# Is tepid sponging more effective than paracetamol at relieving fever in febrile children in hot tropical climates? a mini review

**DOI:** 10.4314/gmj.v55i1.9

**Published:** 2021-03

**Authors:** Samuel Akyirem, Irene F Bossman

**Affiliations:** 1 King's College London, United Kingdom; 2 SDA Nursing and Midwifery Training College Asanta, Ghana; 3 East Sussex NHS Trust, United Kingdom

**Keywords:** Fever, Tepid sponging, Paracetamol, Meta-analysis, children

## Abstract

**Background:**

Childhood fever remains a significant health problem because of the convulsion risk it poses to the child as well as the parental anxiety it provokes. Tepid sponging of such children remains commonplace in tropical climates despite the lack of evidence to support it.

**Objective:**

To evaluate the effectiveness of tepid sponging in hot tropical climates

**Methods:**

NICE systematic review methodology was used. Medline and EMBASE were searched from their inception to date. Eligibility criteria included a) studies of randomised controlled trial (RCT) design b) children aged 2 to 120 months c) the use of tepid sponging alone in one arm and paracetamol in the other arm of the experiment. Eligible studies were critically appraised with NICE risk of bias tool. The outcome of interest was the number of afebrile children 2 hours after intervention. The outcome data from eligible studies were pooled for meta-analysis using random effects.

**Findings:**

Out of the 201 papers retrieved from the electronic search, two studies met the inclusion criteria. The meta-analysis found that tepid sponging was less effective than paracetamol at relieving fever two hours post-intervention (RR=0.25, 95% CI 0.08-0.79]).

**Conclusion:**

Tepid sponging was not effective against fever. There is the need to modify existing local clinical protocols to reflect the new evidence and international guidelines

**Funding:**

None declared

## Introduction

Fever accounts for over 14% of all hospital attendances among children both in primary care 1 and emergency settings.^2^ Childhood fever is even more common in the tropical regions^3^, perhaps due to the higher rate of infectious diseases.^4^ Fever is associated with discomfort in the child, increased risk for convulsions and parental anxiety.^5^ Interventions to reduce fever abound and are usually categorized into physical methods and pharmacological approaches. Physical methods comprise techniques such as tepid sponging which produces its effect of heat loss through conduction, convection and evaporation. In contrast, drug treatments (antipyretics) such as paracetamol produces its effect by inhibiting prostaglandin synthesis which in the long run enhances peripheral vasodilation with attendant heat loss as well as resetting the thermoregulatory center of the hypothalamus to normalcy.

The National Institute of Health and Care Excellence (NICE) strongly recommend against the use of tepid sponging in febrile children in the UK.^6^ Moreover, various systematic reviews evaluating tepid sponging have emphasized the lack of evidence to support its continual use. 7,8 Nonetheless, it has been argued that in hot and/or humid climates, such as the tropical regions, tepid sponging may prove beneficial.^9^,^10^

Indeed, anecdotal evidence from Ghana, a hot tropical country, suggests that tepid sponging remains widely used by caregivers at homes and hospitals perhaps due to its low cost and relative ease of use. Furthermore, in tropical India, up to 75% of mothers continue to sponge their febrile children.^11^ It is, therefore, necessary to evaluate the effectiveness of tepid sponging in these settings by comparing with the most commonly used antipyretic, paracetamol.

A scoping search of extant literature revealed a dearth of reviews that perform a head-to-head comparison of tepid sponging and paracetamol or reviews that focus on sponging in hot tropical climates. The most recent Cochrane Review on tepid sponging demonstrated that sponging was less effective than antipyretics.^8^ However, the review combined studies from both tropical and non-tropical countries. The current systematic review therefore seeks to address the evidence gap by answering the foreground intervention question: “Is tepid sponging more effective than paracetamol at relieving fever in febrile children in hot tropical climates”? using randomized controlled trials (Table 1).

**Table 1:** PICO for the review question

POPULATION (P)	INTERVENTION (I)	COMPARISON (C)	OUTCOME (O)	STUDY DESIGN
**Febrile children** **(2 months to 12** **years)**	Tepid sponging	Paracetamol	Fever	Randomized Controlled trial (RCT)

## Methods

This study was designed as a “mini-review”^12^, conducted in accordance with the NICE methodology guidelines for systematic reviews (NICE, 2012) and reported with PRISMA guidelines. 13 An electronic search was conducted on Medline (1946 to March 23, 2020) and EMBASE (1974 to 2020 week 12) databases. Medline was selected because it possesses a large body of medical literature while EMBASE focuses on drug treatments.

A facet analysis was performed to identify key components of the review question that will inform the search strategy. For each identified facet, index terms, keywords, alternate spelling, and synonyms were generated as shown in table 2. “Physical method” was added as a search term because of the continuous reference to it in the literature. In addition, truncation ($,*), proximity searching and wildcards (?) were used as appropriate to expand the search. Boolean combination (OR/AND) of search terms were performed as appropriate. Details of search strategy is provided in Appendix 1.

**Table 2:** Facet analysis of review question

	POPULATION		INTERVENTION		COMPARISON		OUTCOME
**INDEX TERMS**	Fever OR	**AND**	Hydrotherapy OR	**AND**	Acetaminophen OR	**AND**	(Not used)
**KEYWORDS**	Fever OR		Tepid spong* OR		Paracetamol OR		
	(High adj3 temperature) OR		Tepid bath* OR		Antipyretic* OR		
	Febrile		Spong* OR		Acetaminophen		
			Hydrotherapy OR				
			Physical method*				

Identified studies were then imported into Covidence (https://www.covidence.org) for title and abstract screening. Eligibility criteria applied is summarized in the Table 3. Full texts of potentially eligible studies were then retrieved and read to ascertain their eligibility. The risk of bias of eligible studies were assessed using the NICE risk of bias tool (NICE, 2012). A data extraction form was used to obtain pertinent data from included studies. Data were then summarized and tabulated. Risk ratios (RR) and their corresponding 95% confidence intervals were calculated for the outcome in each study. Data from included studies were pooled and meta analyzed using random effects. Analysis was done with Review Manager v5.5.^14^ To ensure transparency and objectivity in the selection and synthesis of evidence, the two authors independently conducted the search, screening, appraisal and synthesis. No ethical approval was required for this study because of the reliance on secondary published data.

**Table 3:** Inclusion and Exclusion criteria

	Inclusion	Exclusion
**Study design**	Randomized controlled trial, Primary research.	Quasi-experimental studies, non-experimental studies
**Population**	Children aged 2 months to 12 years. Study done in a tropical climate Children with fever (as defined by study)	Children in non-tropical climates, adults
**Intervention**	Tepid sponging or bathing alone	Sponging + other interventions. All composite interventions are excluded
**Comparator**	Paracetamol alone	Paracetamol + other interventions such as sponging
**Outcome** **measure**	Number of afebrile children (as defined by study) 2 hours post intervention	

**Appendix 1:** Search strategy on Medline (1946 to March 23, 2020)

#	Searches	Results
**1**	exp Fever/	42726
**2**	fever.mp.	213000
**3**	febrile.mp.	35696
**4**	pyrexia.mp.	4489
**5**	hyperpyrexia.mp.	1275
**6**	hyperthermia.mp.	35388
**7**	(high adj3 temperature).mp.	42322
**8**	1 or 2 or 3 or 4 or 5 or 6 or 7	308033
**9**	tepid spong*.mp.	34
**10**	tepid massag*.mp.	2
**11**	spong*.mp.	43072
**12**	exp Hydrotherapy/	19880
**13**	hydrotherapy.mp.	2963
**14**	physical method*.mp.	2182
**15**	tepid bath*.mp.	5
**16**	9 or 10 or 11 or 12 or 13 or 14 or 15	65497
**17**	exp Acetaminophen/	17902
**18**	antipyretic*.mp.	7163
**19**	paracetamol.mp.	11425
**20**	acetaminophen.mp.	23899
**21**	17 or 18 or 19 or 20	33979
**22**	8 and 16 and 21	80

## Results

The search on the two electronic databases yielded 201 papers. After removing duplicates 139 were left. A total of 126 studies (mostly observational studies) were found ineligible after title and abstract screening.

**Figure 1:**
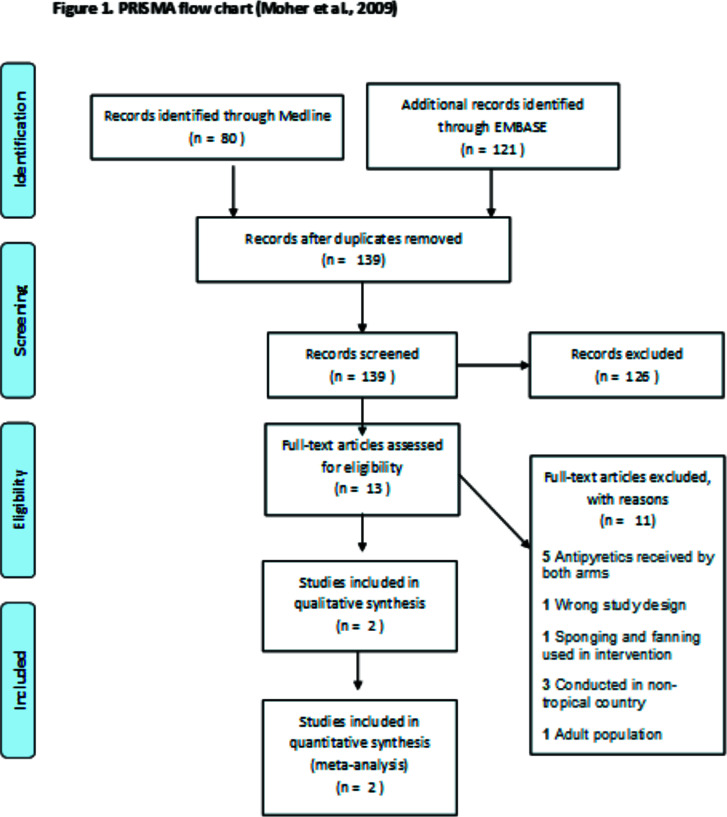
PRISMA flow chart (Moher et al., 2009)

Full texts of the remaining 13 studies were assessed against the pre-set inclusion criteria. Eleven (11) studies were excluded for the following reasons: antipyretic received by both arms^7^,^9^,^14^–^16^, study design not RCT^17^, study conducted outside tropical settings^18^–^20^, use of adult population^21^ and use of other physical method of temperature control^22^. Details of the screening process and reasons for exclusion are presented in Figure 1 and Table 4 respectively.

**Table 4:** Table of exclusion

No.	Study	Reason for exclusion
**1**	Barton et al., 1990	Study conducted in non-tropical country (Turkey)
**2**	Thomas et al., 2009	Study conducted in non-tropical country (USA)
**3**	Bernath et al., 2002	This study was a systematic review (not a primary study)
**4**	Brandts et al., 1997	Patients in both intervention and control groups received other treatment (Quinine) in addition to the antipyretic measures Other forms of physical methods of temperature control such as fanning were used in addition to sponging.
**5**	Khaliq et al., 2019	Both intervention and control groups received paracetamol
**6**	Mahar et al., 1994	Both intervention and control groups received paracetamol
**7**	Newman, 1985	Patients in intervention and control groups received antipyretic medications
**8**	Salgado et al., 2016	Study involved adult participants only
**9**	Sharber, 1997	Both intervention and control groups received paracetamol
**10**	Steele, 1970	Study was conducted in a non-tropical country (USA)
**11**	Thomas et al., 2009	Both intervention and control groups received paracetamol medication

Two peer-reviewed studies, satisfying the eligibility criteria, were finally included in this review.^23^,^24^ The studies had a total of 168 participants (91 males; 77 females). They were of RCT design and performed a head-to-head comparison of sponging and oral paracetamol (15mg/kg). The studies were both conducted in tropical climates i.e. Nigeria^24^ and Malawi^23^ with ambient temperatures ranging from 23°C to 33°C. The studies involved febrile children (38°C to 40°C), aged between 6 to 120 months with an average follow-up time of 2hours post-intervention. Details of the two studies are summarized in Table 6.

**Table 6:** Summary of evidence

Bibliographic reference	Study type	Number of participants	Participant characteristic	Intervention	Settings	Comparison	Length of follow-up	Outcome measures and effect size	Quality assessment	Additional comments
Aluka et al (2013)	RCT	N=88 4 – “rescued” 4 – dropped out	48 males 40 females; IG: age (mean, SD) – 36.23 (28.9) months CG: age (m, SD) – 42.84(32.1) months Initial temp (m, SD) – 38.8 (0.6) °C	Cold water sponging from head-to-toe by mother, caregiver or research assistant for 30mins	Nigeria	A single dose of 15mg/kg paracetamol	2 hours	Number of afebrile children 2 hours post intervention (calculated effect size in table 7). The sponging and paracetamol groups recorded a temp drop of 0.39°C and 1.6°C respectively	Moderate quality	Ambient temperature between 23.0°C and 33.0°C
Agbolosu et al (1997)	RCT	N=80	43 Males, 37 females; IG: age (mean, SD)– 19.1 (12.7) months; CG: age (m, SD) – 17.6 (12.04) months. Initial temp – 38.5 to 40°C diagnosis – 45 URTI, 31 Malaria, 4 Malaria/ URTI;	Head-to-toe sponging with water (28°C to 34°C), leaving a thin layer of water on the body. Repeated until temperature fell below 38.5°C	Malawi	A single dose of 15mg/kg paracetamol	2 hours	Number of afebrile children after 2 hours (calculated effect size in table 7) The sponging and paracetamol groups recorded a temp drop of 0.75°C and 1.83°C respectively	Moderate quality	Ambient temperature 21°C to 32°C

IG – intervention group; CG: control group; temp – temperature; SD – standard deviation; RCT – randomized controlled trial; m – mean

### Risk of bias assessment

The two included studies reported the use of randomization to allocate participants to treatment groups. Aluka et al^24^ gave details of their sequence generation, allocation of patients to groups using balloting and how allocation concealment was enforced. In contrast, Agbolosu et al^23^ cited the use of block randomization but provided no details of how this was realized. However, unlike Aluka, Agbolosu rightly compared the diagnosis and body surface area of patients in the intervention/control groups at baseline and found no significant differences (i.e. intervention and control groups were matched based on diagnosis). Aluka et al. study should have reported such factors as they are potential confounders and have been reported to influence the effects of sponging.^15^ Notwithstanding, the intervention/control groups in each study had comparable baseline data. Hence, the included papers had low risk of selection bias (Table 5).

**Table 5:** Risk for bias assessment

	Selection bias	Performance bias	Attrition bias	Detection bias
**Aluka et al (2013)**	Low risk	Low risk	Unclear	Low risk
**Agbolosu et al (1997)**	Low risk	Low risk	Low risk	Low risk

There was a low risk of performance bias for the 2 studies. The nature of the treatments made it impossible to blind subjects and interventionists. The absence of blinding may be less concerning for a study with an objective outcome measure such as temperature.

Also, it was not clear who delivered the intervention in the Agbolosu study. In the paper by Aluka *et al*,^24^ sponging was performed by mothers, nurses or research assistants.

Disparities in the competencies of the various interventionists could have influenced how well the intervention was administered and subsequently, the outcome. Both papers however reported that comparison groups received same care apart from the intervention being studied.

No attrition was recorded in Agbolosu's study and all subjects were accounted for in the analysis. Hence the risk of attrition bias was low. Aluka et al on the other hand, reports that four children in the sponging group needed further treatment and hence were “rescued” from the study and excluded from the analysis (i.e. no intention-to-treat analysis performed). Moreover, no reason was given for the drop out of four additional participants. The study did not provide the characteristics of these eight children to enable the reader to determine if they differ from those that stayed on. The risk of attrition bias for this study was therefore unclear.

Risk of detection bias was low for the two studies. Primary and secondary outcomes were clearly defined in both studies. Reliable instruments were also used. Aluka performed test-retest reliability tests of their mercury thermometer prior to the study. Similarly, rectal and axillary temperatures were concurrently taken in the Agbolosu study to ensure accurate and reliable temperature measurements.

In both studies, it is unclear if the same thermometer was used to assess temperature in the control and intervention groups, paving way for a potential differential bias. Both papers were unclear about blinding of investigators and outcome assessors. This may be of less impact for an objective outcome 25.

Overall, the Agbolosu study^23^ and the Aluka study^24^ were of moderate quality. The quality assessment identified no major bias that could compromise the internal validity of the studies.

### Synthesis of results

Results from individual studies are presented in Table 7. Agbolosu et al. found that two hours after intervention, significantly fewer children remained afebrile in the sponging group than in the paracetamol group (p<0.001). The risk of being afebrile was therefore 61% lower in the sponging than the paracetamol group (RR = 0.39, 95% Confidence Interval [CI] 0.26–0.59). Moreover, Agbolosu et al.^23^, reported a mean temperature drop of 0.75°C (from 39.3°C) and 1.83°C (from 39.1°C) in the sponging and paracetamol groups respectively, Similar results were seen in the Aluka study^24^ showing an 87% less risk of a sponged child becoming afebrile (RR= 0.13, 95% CI: 0.05–0.34, p<0.001). In addition, a 0.39°C (from 38.84°C) and 1.6°C (from 38.72°C) decline in temperatures were observed in the sponging and paracetamol groups respectively.

**Table 7:** Outcome table – Number of afebrile children 2 hours post-intervention

Study	Events in tepid sponging group/total	Events in paracetamol group/total	Risk Ratio (95% confidence interval)	P-value	Interpretation (statistical and clinical significance)
**Agbolosu et al**	15/40	38/40	0.39 (0.26 – 0.59)	<0.001	Statistically significant Strong clinical significance regarding the ineffectiveness of tepid sponging
**Aluka et al**	4/38	33/42	0.13 (0.05 – 0.34)	<0.001	Statistically significant Strong clinical significance regarding the ineffectiveness of tepid sponging

**Figure 2:**
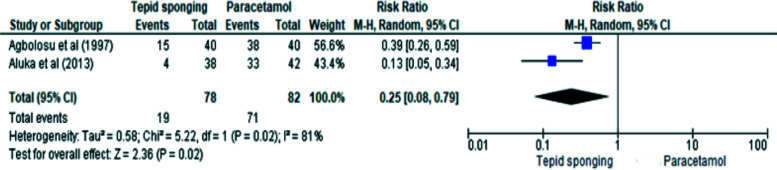
Meta-analysis and Forest plot

Data from the two studies were pooled for meta-analysis using random effects (Figure 2). The similar but unidentical effect sizes observed in the two studies informed the use of the random effect model.^26^

Overall, 160 children (78 tepid sponging group and 82 paracetamol group) were included in the analysis. The Agbolosu study had more precise results (narrow 95% CI) and contributed slightly more weight (56.6%) to the meta-analysis than the Aluka study (wider 95% CI and Weight = 43.4%). The meta-analysis revealed that children who were sponged were 75% less likely to be afebrile two hours after intervention compared to those who received paracetamol (RR=0.25, 95% CI 0.08–0.79]). The pooled statistic was imprecise due its large confidence interval. A high level of heterogeneity (I^2^=81%, p=0.02) was observed. This may stem from the small number of pooled studies (n=2) and the small sample size of included studies. 27 From the GRADE assessment (Table 8), the overall strength of the pooled evidence was adjudged moderate.

**Table 8:** GRADE assessment (Generated using https://www.GradePro.com)

Quality assessment							No. of patients		Effects		Certainty	Importance
Number of studies	Design	Risk of bias	Inconsistency	Indirectness	Imprecision	Publication bias	Tepid sponging	Paracetamol	Relative (95% CI)	Absolute (95% CI)		
Number of afebrile children (follow up: median 2 hours)												
2	Randomized trial	Not serious^a^	Not serious^b^	Not serious	Serious^c^	Not detected	19/78 (24.4%)	71/82 (86.6%)	RR – 0.25 (95% CI 0.08–0.79)	649 fewer per 1000 (from 797 fewer to 182 fewer)	+++ (MODERATE)	Important

a Risk of bias for the 2 studies were low except for the Aluka et al study which had unclear risk of attrition bias. Hence, a decision was made not to downgrade.

b Heterogeneity score for pooled data was high (I^2^=81%) but this anomaly may be explained by the few numbers of included studies (n=2). Hence, a decision was made not to downgrade.

c Confidence interval for pooled risk ratio (RR) was too wide and imprecise. This observation may however stem from the use of the random effects in the metaanalysis 27 and the small number of studies.

## Discussion

This review sought to search, critically appraise and synthesize existing evidence comparing sponging to paracetamol to determine the comparative efficacy of these treatment options at relieving childhood fever in tropical climates.

The study found that 2 hours after intervention, significantly fewer children remained afebrile in the sponging group as compared to the paracetamol group. Sponging was therefore ineffective at relieving fever in tropical climates.

This finding is in consonance with existing literature and clinical guidelines.^6^,^28^ In a review by Lim et al (2018), sponging was found to be effective only in the short-term but conferred no benefit to patients in the long-term (after 2hrs). A similar review by Meremikwu reached same conclusion 28.

In hot climates, heat loss to the environment may not readily occur following antipyretic use. 7 The use of external cooling may facilitate the heat loss process. A dual therapy of sponging and antipyretics may prove beneficial as suggested by some studies. 28 Further empirical studies evaluating these dual therapies should be conducted in tropical settings to provide evidence to support or refute their use.

Though sponging mono-therapy has been found to be less effective, the absence of locally relevant clinical guidelines in tropical settings has contributed to its continual use. 29 In Ghana, for instance, nurses are still trained to perform sponging in contrast to WHO's recommendations.^30^ There is therefore the need to design locally relevant clinical guidelines to inform best practices in these settings.

A major strength of this review was the involvement of two independent reviewers along each step of the review process to ensure objectivity in evidence synthesis. Nonetheless, the review had some limitations. Important papers might have been missed since the search was done on only 2 databases. Furthermore, only two papers were included in the synthesis making it difficult to draw a firm conclusion on the topic.

Some inconsistencies in the definition of “fever” were noted, with each study arbitrarily setting its own limit for normal temperature. This might have influenced the number of afebrile children observed in each study. Future studies should therefore adhere to standardized definitions of fever. 6

## Conclusion

This review found that tepid sponging was less effective than paracetamol. Considering the side effects of sponging reported in other studies 8, the quality of the evidence from this review, and the low cost and ease of use of sponging, a strong recommendation against the use of tepid sponging alone in the treatment of fever in tropical settings can be made. Though combination treatments may be beneficial, further evidence is required.
